# Editorial: Recent advances in *Campylobacter* research

**DOI:** 10.3389/fmicb.2025.1646828

**Published:** 2025-07-08

**Authors:** Stuart A. Thompson, Nicolae Corcionivoschi, Odile Tresse, Ozan Gundogdu

**Affiliations:** ^1^Department of Medicine, Division of Infectious Diseases, Augusta University, Augusta, GA, United States; ^2^Bacteriology Branch, Veterinary Sciences Division, Agri-Food and Biosciences Institute, Belfast, United Kingdom; ^3^Nantes Université/INRAE, UMR 1280, PhAN, Nantes, France; ^4^Faculty of Infectious and Tropical Diseases, London School of Hygiene and Tropical Medicine, London, United Kingdom

**Keywords:** biofilm, *Campylobacter jejuni*, *Campylobacter* coli, *Campylobacter* concisus, *Campylobacter* fetus, Antibiotic resistance (ABR), microbiota

*Campylobacter* spp. are responsible for more than 100 million cases of human disease each year. While the majority of cases result from infection with *C. jejuni* and *C. coli*, other *Campylobacter* species such as *C. concisus* are increasingly recognized as being responsible for disease pathogenesis. Additionally, species such as *C. fetus* are important veterinary pathogens. As with many pathogens, antibiotic resistance in *Campylobacter* is becoming increasingly problematic, leading to these bacteria being classified as high-level threats by the U.S. Centers for Disease Control (CDC) and the World Health Organization (WHO). However, despite decades of study, the mechanisms by which these bacteria cause illness are not fully understood, and no effective control strategies for animal reservoirs exist. In this Special Topic in Frontiers in Microbiology, “*Recent Advances in Campylobacter research*,” features 10 manuscripts that explore various aspects of *Campylobacter* antibiotic resistance, host colonization, and pathogenesis.

Cho et al. used RNA-Seq to evaluate the responses of *C. jejuni* to antibiotic tolerance induced by ciprofloxacin and tetracycline. The transcriptional response was surprisingly broad, and encompassed changes in numerous genes of various functional categories that presumably promote survival upon exposure to antibiotics. In particular, mutational studies showed that protein chaperones facilitate cellular survival by managing protein disaggregation.

García-Fernández et al. performed a study on a collection of Italian isolates of *C. jejuni* and *C. coli*, using Whole-Genome Sequence (WGS) and Multilocus Sequence Typing (MLST) to evaluate the prevalence of antibiotic resistance and virulence determinants. The authors found widespread antibiotic resistance in both species. Furthermore, virulence determinants were more highly represented in specific MLST types, suggesting the potential of WGS to identify strains of greater clinical significance.

A study by Deforet et al. explored *Campylobacter* antibiotic resistance. Aminopenicillin resistance is associated with a point mutation in the promoter for the chromosomal β-lactamase gene *bla*_*OXA*61_; however, this can generally be overcome by combination treatment with clavulanic acid. While resistance to amoxicillin-clavulanic acid is rare, the authors identified three *C. coli* strains with amoxicillin-clavulanic acid resistance. A combination of WGS and mass spectrometry (MS) was used to identify the mechanism of this resistance as a second promoter mutation that results in increased *bla*_*OXA*61_ expression.

Concerning therapeutics against *Campylobacter*, Deblais et al. targeted the twin-arginine translocation (Tat) system, which is responsible for *C. jejuni* formate utilization but is not present in mammals and chickens. Inhibition of this system, therefore, may prevent *C. jejuni* colonization. Because the Tat system also mediates CuSO_4_ resistance by *C. jejuni*, the authors identified small-molecule inhibitors of the Tat pathway by selecting for increased susceptibility to CuSO_4_. The identified small-molecule inhibitors were non-toxic to Caco-2 cells and reduced *Campylobacter* colonization in chicks, suggesting their utility as a control strategy.

One major impediment to a better understanding of *C. jejuni* pathogenesis is the lack of effective mammalian colonization models, due in part to the inhibitory effect of the intestinal microbiota of mouse strains on *Campylobacter* colonization. To gain a greater understanding of this issue, Shayya et al. used a targeted metabolomics approach to define characteristics associated with colonization resistance. The microbiota of mice with colonization resistance contained greater abundances of lactobacilli and Mouse Intestinal Bacteroides, and lower abundances of enterobacteria, enterococci, and *Clostridium coccoides* group. This microbial community structure was accompanied by elevated levels of antimicrobial bile acids and fatty acids, and a reduced abundance of amino acids that are essential for *C. jejuni* growth, thus providing an explanation for colonization resistance.

To further develop mouse models and to study therapeutic interventions against *C. jejuni* infection, Mousavi et al. used human microbiota-associated IL-10^−/−^ mice, which developed symptoms of acute campylobacteriosis. Administration of carvacrol (alone or in combination with deferoxamine, deoxycholate, and 2-fucosyl-lactose) resulted in a reduced ileal load of *C. jejuni*, along with a decrease in diarrhea and histopathological/inflammatory responses. These compounds may therefore represent a promising therapeutic approach as opposed to antibiotic therapy.

To examine the interactions between *C. jejuni* and other bacteria, Dreyer et al. used a data-independent acquisition mass spectrometry (DIA-MS) approach to determine changes in the whole-cell proteome of *C. jejuni* upon co-colonization with three Gram-positive *bacterial species*, and exposure to the bile acid deoxycholate (DCA). All three co-incubation scenarios induced large-scale changes to the *C. jejuni* proteome and allowed the identification of a core set of 54 proteins that comprised a common co-incubation response. Although the response to DCA was substantially larger, the co-incubation and DCA responses partially overlapped, and data suggested a synergistic response to the different stimuli.

In contrast to its fastidious nature in laboratory culture and its sensitivity to atmospheric oxygen, *C. jejuni* can survive in a number of challenging environments. One aspect of its biology that allows it to do so is its ability to form protective biofilms, which are cells encased in an Extracellular Matrix (ECM). Pavlinjek et al. described the physical and chemical methods for isolating this ECM. The isolated ECM varied greatly using the different methods, but was rich in protein, polysaccharides, and extracellular DNA. These methods can be used to select the appropriate ones for downstream analytical experiments.

Luk et al. examined the gastric pathogenicity of *C. concisus*, using cultured gastric epithelial AGS cells. Exposure to *C. concisus* induced several changes in AGS cells, including the induction of IL-8, actin polymerization, and caspase 3/7. Significantly, *C. concisus* also elicited an increase in *CYP1A1* gene expression; increased *CYP1A1* expression is associated with poorer survival in gastric cancer patients. Together, these data suggest that *C. concisus* may induce gastric inflammation and could affect gastric cancer prognosis.

Finally, Ong et al. used WGS to gain further insight into the veterinary pathogen *C. fetus* subsp. *fetus* (four genomes) and *C. fetus* subsp. *venerealis* (five genomes). Despite host differences between the two subspecies, they have been difficult to distinguish from each other. While the genomes of the two subspecies were remarkably similar in this study, within this conserved framework were several single-nucleotide polymorphisms that can be used to distinguish the two subspecies.

Together, the manuscripts published in this Research Topic represent important advances in various areas of *Campylobacter* research.

